# Randomized clinical trial comparing liver resection with and without perioperative assessment of liver function

**DOI:** 10.1002/bjs5.81

**Published:** 2018-06-14

**Authors:** M. Stockmann, F. W. R. Vondran, R. Fahrner, H. M. Tautenhahn, J. Mittler, H. Bektas, M. Malinowski, M. Jara, I. Klein, J. F. Lock, on behalf of the Collaborative Fast‐track Liver Study Group, J. Pratschke, S. Chopra, G. Hunold, J. Klempnauer, K. Timrott, S. Cammann, M. D. Jäger, B. Kettler, M. Kleine, W. Knitsch, U. Kulik, F. Oldhafer, W. Ramackers, C. Schumacher, L. Kousoulas, U. Settmacher, A. Nikolic, A. Bauschke, M. Gampe, H. Scheuerlein, A. Koch, V. Mategakis, L. Mutwol, U. Schöne, F. Uteß, F. Rauchfuß, M. Bartels, J. Broschewitz, J. Bucher, K. Mankel, G. Wiltberger, C. Benzing, L. Feldbrügge, M. Schmelzle, M. Schönberg, K. Semmling, H. Lang, S. Heinrich, T. Huber, K. Alghdban, M. Kochergin, C. T. Germer, F. Anger, U. Steger

**Affiliations:** ^1^ Department of General, Visceral and Transplantation Surgery Charité – Universitätsmedizin Berlin Germany; ^2^ Department of General, Visceral and Transplant Surgery Hannover Medical School Hannover Germany; ^3^ Department of General, Visceral and Vascular Surgery University of Jena Jena Germany; ^4^ Department of Visceral, Transplant, Thoracic and Vascular Surgery University Hospital Leipzig Leipzig Germany; ^5^ Department of Hepatobiliary and Transplantation Surgery Johannes Gutenberg University Mainz Germany; ^6^ Department of General, Visceral, Vascular and Paediatric Surgery University Hospital of Würzburg Würzburg Germany; ^7^ Department of General, Visceral and Vascular Surgery Evangelisches Krankenhaus Paul Gerhardt Stift Lutherstadt Wittenberg Germany; ^8^ Department of General, Visceral and Oncological Surgery Bremen Mitte Clinic Bremen Germany; ^9^ Department of General, Visceral, Vascular and Paediatric Surgery University of Saarland Homburg Germany; ^10^ Charité–Universitätsmedizin Berlin Berlin Germany; ^11^ Hannover Medical School Hannover Germany; ^12^ University of Jena Jena Germany; ^13^ University Hospital Leipzig Leipzig Germany; ^14^ Johannes Gutenberg University Mainz Germany; ^15^ University Hospital of Würzburg Würzburg Germany

## Abstract

**Background:**

Liver function tests may help to predict outcomes after liver surgery. The aim of this study was to evaluate the clinical impact on postoperative outcome and patient management of perioperative liver function testing using the LiMAx^®^ test.

**Methods:**

A multicentre RCT was conducted in six academic liver centres. Patients with intrahepatic tumours scheduled for open liver resection of at least one segment were eligible. Patients were randomized to undergo additional perioperative liver function tests (LiMAx^®^ group) or standard care (control group). Patients in the intervention arm received two perioperative LiMAx^®^ tests, one before the operation for surgical planning and another after surgery for postoperative management. The primary endpoint was the proportion of patients transferred directly to a general ward. Secondary endpoints were severe complications, length of hospital stay (LOS) and length of intermediate care/ICU (LOI) stay.

**Results:**

Some 148 patients were randomized. Thirty‐six of 58 patients (62 per cent) in the LiMAx^®^ group were transferred directly to a general ward, compared with one of 60 (2 per cent) in the control group (P < 0·001). The rate of severe complications was significantly lower in the LiMAx^®^ group (14 per cent versus 28 per cent in the control group; P = 0·022). LOS and LOI were significantly shorter in the LiMAx^®^ group (LOS: 10·6 versus 13·3 days respectively, P = 0·012; LOI: 0·8 versus 3·0 days, P < 0·001).

**Conclusion:**

Perioperative use of the LiMAx^®^ test improves postoperative management and reduces the incidence of severe complications after liver surgery. Registration number: NCT01785082 ( https://clinicaltrials.gov).

## Introduction

Liver resection is the treatment of choice for most hepatic malignancies and has become a safe and effective surgical procedure^1^. Major resection and underlying hepatic injury, however, increase the risk of postoperative liver failure with consecutive morbidity and mortality. The lack of suitable diagnostic tests to predict the individual risk of postoperative liver failure led to the development of the LiMAx^®^ test (Humedics, Berlin, Germany), a [^13^C]methacetin‐based metabolic liver function capacity test^2^. Residual liver capacity determined by LiMAx^®^ is one of the major factors influencing postoperative complications^2^.

Since the first experimental application of the LiMAx^®^ test in 2004, its diagnostic accuracy and clinical potential have been shown in several clinical fields, including surgery^2^, ^3^, ^4^, ^5^, ^6^, ^7^, transplantation medicine^8^, ^9^, ^10^, intensive care^11^, ^12^, ^13^ and hepatology^14^, ^15^, ^16^. The LiMAx^®^ test accurately determines liver function before^2^, ^3^, ^4^, ^5^, ^6^ and after^17^, ^18^, ^19^ liver surgery. A retrospective analysis^4^ showed a striking decrease of postoperative liver failure and postoperative liver failure‐related mortality following implementation of the LiMAx^®^ algorithm. Randomized trials evaluating the actual clinical impact of the LiMAx^®^ test application on postoperative outcome after liver surgery have not yet been performed. The aim of this RCT was to address the clinical impact of perioperative liver function assessment by the LiMAx^®^ test on early postoperative outcome and patient management after open liver surgery.

## Methods

### Study design

This study was a phase III, multicentre, two‐arm, parallel‐group, open‐label RCT. Patients were recruited from six German academic centres specialized in complex liver surgery. The trial followed the ethical guidelines of the 1975 Declaration of Helsinki and the CONSORT 2010 guidelines^20^. The protocol was approved by the responsible ethics committee and approved by the German Federal Institute for Drugs and Medical Devices. The trial was registered as NCT01785082 (http://clinicaltrials.gov).

### Participants

Patients aged 18 years or more with benign or malignant intrahepatic tumours scheduled for open liver resection of at least one segment were eligible; they were included after written informed consent had been obtained. Contrast‐enhanced three‐phase thin‐layer CT or high‐quality MRI of the liver within the past 6 weeks was required for resection planning. Exclusion criteria were expected vascular or biliary anastomosis, history of previous liver resection, known liver cirrhosis or severe fibrosis, and severe co‐morbidities requiring postoperative telemetry. In the study protocol, criteria were defined that allowed for replacement of participants if the planned procedure was not performed owing to, for example, advanced tumour stage or tumour extension to other solid organs.

### Randomization

Before randomization, surgeons determined each patient's postoperative care, including the indication of postoperative transfer to a telemetry unit (providing continuous cardiac, haemodynamic and respiratory monitoring, typically on the intermediate care unit (IMCU) or ICU). The assignment was documented with date and time, and signed by the responsible surgeon. Thereafter, patients were randomized either to the intervention arm (LiMAx^®^ group) or to the standard‐care arm (control group) in a ratio of 1 : 1 at each centre. The randomization was stratified for each centre and each preoperative assignment (postoperative care on general ward *versus* telemetry on the IMCU/ICU) using sealed, sequentially numbered, randomization envelopes provided by IFS (Institute for Applied Research and Clinical Studies), Göttingen, Germany. Patient enrolment and randomization were performed by trained study investigators at the study centres.

### Intervention

Two LiMAx^®^ test assessments were performed in patients in the intervention group. The first test was done the day before surgery for individual surgical planning. The resection strategy and intraoperative procedures were adopted before surgery according to the LiMAx^®^ decision tree algorithm for hepatectomy^3^. This algorithm stratifies the risk of postoperative liver failure according to the preoperative LiMAx^®^ test result and the future remnant liver volume. Major resections up to hemihepatectomy can be performed safely when the LiMAx^®^ test shows a normal value (more than 315 μg per kg per h). In patients with impaired liver function or extended resections, individual volume–function analysis by CT or MRI‐based liver volumetry of the residual liver volume and analysis can predict residual liver function.

The second LiMAx^®^ test was done within 6 h after skin closure in the recovery room to determine the individual patient's postoperative management. If the LiMAx^®^ value was greater than 150 μg per kg per h, the patient was eligible for primary postoperative transfer to a general ward, omitting continuous monitoring. The cut‐off value was chosen according to a previous study^3^, which indicated a very low risk of complications when the postoperative LiMAx^®^ value was above 150 μg per kg per h. If non‐hepatic conditions required telemetry (postoperative bleeding, haemodynamic instability, respiratory insufficiency, not sufficiently awake and responsive, no satisfactory level of analgesia) during the stay in the recovery room, patients were transferred to the IMCU/ICU. If the LiMAx^®^ value was 150 μg per kg per h or less, patients were generally transferred to the IMCU/ICU.

In the control group, perioperative management followed standard clinical care without performing a LiMAx^®^ test. These patients were transferred to the respective ward, based primarily on the surgeon's preoperative assignment before randomization. Patients who had been assigned to a general ward but developed a condition requiring telemetry were transferred to the IMCU/ICU.

The applied operative techniques and surgical instruments were neither defined by the study protocol nor influenced by the study arm. All resections were performed by experienced and specialized liver surgeons in high‐volume centres. No additional LiMAx^®^ tests were allowed in either study arm.

### LiMAx^®^ test

Breath tests provide an elegant way to measure *in vivo* metabolic functions using enzyme‐specific ^13^C‐labelled substrates. The most commonly applied substrate for determination of liver function has been methacetin^21^. [^13^C]methacetin is administered and specifically metabolized by the microsomal cytochrome P450 1A2 enzyme in the liver. Consequently, the emerging [^13^C]carbon dioxide is released into the bloodstream and exhaled, leading to an altered ^13^CO_2_/^12^CO_2_ ratio in the breath. This change can be determined by various analytical devices to provide a parameter of the cytochrome P450 1A2‐dependent methacetin conversion rate.

The methacetin breath test was developed in the 1970s, and its diagnostic potential has been described in multiple studies^22^, ^23^, ^24^. The LiMAx*®* test enables the intravenous administration of [^13^C]methacetin and continuous real‐time breath analysis at the bedside. Its general principles^2^ and the safety of the test^2^ ^3^, ^8^ ^21^, including intravenous [^13^C]methacetin administration, have been shown previously.

Sterile [^13^C]methacetin solution (Humbedics, Berlin, Germany) was administered intravenously at a dose of 2 mg/kg bodyweight. Breath analysis was performed using a novel CE‐certified medical device (FLIP®; Humedics). The entire exhaled breath is collected by a specific face mask (Humedics) and transferred through the FLIP^®^ device for quantitative real‐time determination of ^12^CO_2_ and ^13^CO_2_ concentration using a quantum cascade laser^25^ ^26^. The LiMAx^®^ test result (given in micrograms of substrate metabolism per h, normalized to bodyweight) is calculated by the device and provided within 20 min to a maximum of 60 min after substrate administration. The normal value was defined as greater than 315 μg per kg per h in a previous study of healthy controls^27^.

### Study endpoints

The primary endpoint was the proportion of patients who could be safely transferred from the recovery room to a general ward. The accuracy of the primary postoperative allocation to a general ward was evaluated by the following criteria: no transfer to the IMCU/ICU after transfer to a general ward and regular discharge on postoperative day 30 at the latest (true positive). Patients primarily allocated to the IMCU/ICU (LiMAx^®^ value of 150 μg per kg per h or less in the intervention arm and preoperative assignment in the control arm) were reviewed retrospectively by a group of three LiMAx®‐blinded, study‐independent ICU experts. Only when these assessors unanimously confirmed the medical indication for each postoperative IMCU/ICU transfer based on their clinical experience was the transfer rated retrospectively as appropriate (true negative). The decision of the assessors was recorded for each reviewed patient.

Secondary study endpoints included the proportion of patients who developed posthepatectomy liver failure (PHLF), graded according to Rahbari *et al*.^28^, and postoperative complications, graded according to Clavien–Dindo^29^. Complications of grade IIIa and above were considered severe. Additional endpoints were length of hospital stay (LOS) and length of IMCU/ICU (LOI) stay.

### Sample‐size calculation

Sample‐size calculation was *a priori* performed with SAS^®^ 9.2/Proc Power (SAS Institute, Cary, North Carolina, USA) and the determination of exact confidence intervals was performed with SAS^®^ 9.2/Proc Freq/Option Binomial (Exact). Data applied for this calculation were derived from a retrospective analysis of 673 liver resections performed in 2005–2007 at Charité – Universitätsmedizin Berlin. Of these patients, 156 underwent postoperative LiMAx^®^ tests, of whom 61 (39·1 per cent) had a LiMAx^®^ value above 150 μg per kg per h. The general ward indication was assumed for 37·0 per cent of patients in the LiMAx^®^ arm *versus* 4·3 per cent in the control arm, based on the retrospective analysis. This reflects the rather conservative strategy in all participating centres of monitoring most patients after open liver resection. To substantiate a significant group difference with a two‐sided test at a level of α = 0·05 with greater than 90 per cent power, 31 patients were required in each group. It was calculated that 60 patients were required in the LiMAx^®^ group to predict the proportion of false‐positive test results with sufficient precision. Thus, a total of 120 patients were planned to be enrolled, 60 in each study arm.

### Statistical analysis

All randomized patients were considered for analysis of baseline characteristics. Replaced patients (those in whom resection was not performed for reasons not related to the study) were not considered for the efficacy analysis. Patients in the LiMAx^®^ group with missing postoperative LiMAx^®^ values were excluded, as this value was the decision parameter for postoperative management. Percentages and *P* values are based on subjects with evaluable data. χ^2^ test or Fisher's exact test was applied to compare the performed surgical procedures in the two study arms. A centre‐stratified Cochran–Mantel–Haenszel test was used to evaluate differences between the groups. Furthermore, the exact 95 per cent Pearson–Clopper c.i. was calculated for the rate of severe complications in each group (grade IIIa or above according to the Clavien–Dindo classification^29^). Kaplan–Meier estimates were calculated for each study arm and the results were compared by the centre‐stratified log rank test to evaluate LOI stay as well as LOS. LOI stay was calculated in days from the date of IMCU/ICU discharge minus the date of surgery, and LOS was calculated from the date of hospital discharge minus the date of surgery. Patients who were still in the IMCU/ICU on postoperative day 30 were censored. If a patient was not discharged from the hospital by postoperative day 30, LOS was set to 30 days and the patient was treated as censored in the analysis. Patients who died were treated as censored from the date of death.

Statistical analysis was performed using SAS^®^ version 9.2 (SAS Institute, Cary, North Carolina, USA). *P* < 0·050 was considered statistically significant.

## Results

**Figure 1:**
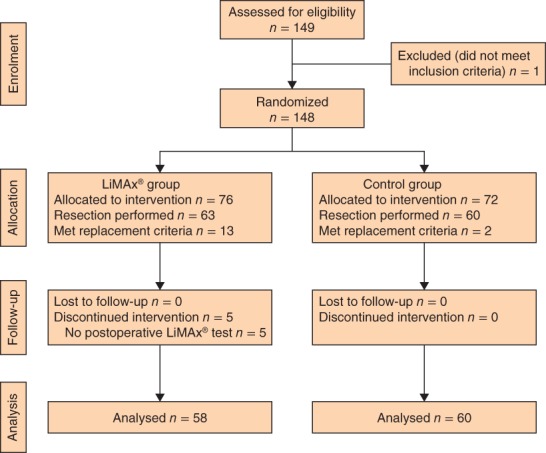
CONSORT diagram for the study

A total of 149 patients were assessed for eligibility between January 2013 and September 2015 (*Fig*. 1). One patient was excluded due to a screening failure (previous liver resection) before surgery. Some 141 patients (95·3 per cent) were planned for postoperative transfer to the IMCU/ICU, and the remaining seven patients (4·7 per cent) were planned for direct postoperative transfer to a general ward. These assignments reflected the conservative standard‐care patient management after open liver resection in all participating centres. Of the seven patients allocated for direct postoperative transfer to the general ward, four were randomized to the LiMAx^®^ group and three to the control group.

Twenty‐five randomized patients (13 in the LiMAx^®^ group and 12 in the control group) were replaced in accordance with the study protocol. Criteria were: surgery not performed/postponed (7 patients), intraoperative change of procedure due to advanced tumour disease (16), and extension of operative procedure to thoracic or other abdominal organs (2). Five patients in the LiMAx^®^ group were excluded because no postoperative LiMAx^®^ test had been performed. Thus, a total of 118 patients, 58 in the LiMAx^®^ group and 60 in the control group, were eligible for analysis.

### Demographics and surgical procedures

Baseline characteristics are shown in Table 1. The majority of patients had surgery for colorectal liver metastases. The most frequent co‐morbidity was hepatic steatosis. Types of operation were comparable in the two groups (Table 2), indicating that the surgical technique employed had not been influenced by the study. In the LiMAx^®^ group, mean(s.d.) preoperative and postoperative LiMAx^®^ values were 437(142) and 241(80) μg per kg per h respectively.

**Table 1:** Baseline characteristics

	LiMAx^®^ group (n = 76)	Control group (n = 72)
Mean(s.d.) age (years)	59·5(13·2)	56·2(14·6)
Sex ratio (M : F)	44 : 32	39 : 33
Weight (kg)	n = 76	n = 70
Mean(s.d.)	78·7(16·6)	80·1(16·8)
Median (range)	77 (53–142)	80 (43–113)
Height (cm)	n = 76	n = 70
Mean(s.d.)	170·6 (8·6)	171·8 (9·0)
Median (range)	171 (150–186)	173 (150–189)
Indication for surgery		
Hepatocellular carcinoma	13 (17)	8 (11)
Cholangiocelluar carcinoma	9 (12)	5 (7)
Liver metastasis, colorectal	27 (36)	34 (47)
Liver metastasis, melanoma	3 (4)	1 (1)
Adenoma (liver)	4 (5)	4 (6)
Focal nodular hyperplasia	2 (3)	3 (4)
Other	7 (9)	12 (17)
Other liver metastasis	11 (14)	5 (7)
Relevant concomitant disease		
Chronic hepatitis B	1 (1)	1 (1)
Chronic hepatitis C	0 (0)	0 (0)
Non‐alcoholic steatohepatitis	3 (4)	1 (1)
Autoimmune hepatitis	0 (0)	0 (0)
Primary biliary cirrhosis	0 (0)	0 (0)
Primary sclerotic cholangitis	0 (0)	0 (0)
Liver cirrhosis	0 (0)	0 (0)
Hepatic steatosis	14 (18)	10 (14)
Previous surgery or therapy		
Yes	70 (92)	69 (96)
No	6 (8)	3 (4)
Intake of medication 30 days before inclusion		
Yes	57 (75)	55 (76)
No	19 (25)	17 (24)

Values in parentheses are percentages unless indicated otherwise.

**Table 2:** Surgical techniques used in patients with evaluable data

	LiMAx^®^ group (n = 58)	Control group (n = 58)	P *
Right hemihepatectomy	11 (19)	10 (17)	0·810
Right extended hemihepatectomy	3 (5)	7 (12)	0·186
Left hemihepatectomy	8 (14)	8 (14)	1·000
Left extended hemihepatectomy	2 (3)	3 (5)	1·000
Left lateral resection	1 (2)	0 (0)	1·000
Segmental resection	22 (38)	19 (33)	0·560
Other resection	11 (19)	11 (19)	1·000

Values in parentheses are percentages.

* χ^2^ test or Fisher's exact test.

### Postoperative management

In the LiMAx^®^ group, 52 of 58 patients (90 per cent) had a postoperative LiMAx^®^ value above 150 μg per kg per h, indicating sufficient liver capacity. Thirty‐six patients (62 (95 per cent c.i. 48 to 75) per cent) were transferred directly to a general ward, compared with one patient (2 (0 to 9) per cent) in the control group (P < 0·001). All patients primarily transferred to a general ward remained there until discharge within 30 days after surgery.

Despite having an LiMAx^®^ value above 150 μg per kg per h, 16 of these 52 patients (31 per cent) were transferred to the IMCU/ICU, in line with the non‐hepatic criteria. Only six patients in the LiMAx^®^ group had a postoperative LiMAx^®^ value of 150 μg per kg per h or less, and were primarily transferred to the IMCU/ICU according to the protocol.

In the control group, three patients had been considered as potentially suitable for transfer to a general ward. Two of these patients were assessed after surgery by the responsible physician as not suitable for transfer to a general ward. Thus, only one patient in the control group originally planned for transfer to a general ward was finally transferred to a general ward. This patient stayed on the general ward until regular discharge within 30 days.

In the group with a postoperative LiMAx^®^ value of 150 μg per kg per h or less, the three external assessors did not confirm the need for postoperative telemetry in four patients. In the control group, 57 of 60 patients had a preoperative assignment to the IMCU/ICU, but this transfer decision was not deemed necessary by the external assessors for 24 of them (42 per cent).

### Length of stay

**Figure 2:**
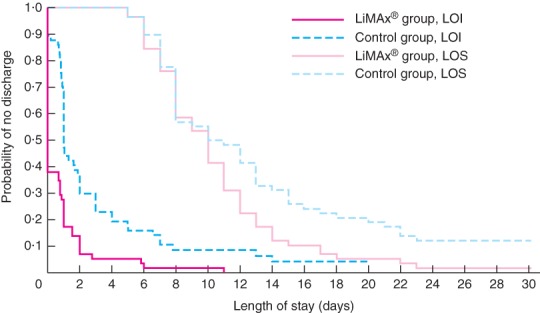
Kaplan–Meier curves for total length of stay in the intermediate care unit or ICU (LOI stay) and length of hospital stay (LOS) by study arm. LOI stay: P < 0·001; LOS: P = 0·012 (log rank test)

Time‐to‐event curves for LOI were evaluated for 58 patients in the LiMAx^®^ group and 57 in the control group (Fig. 2). Three of the control group patients were censored (patient still in IMCU/ICU at postoperative day 30). Mean LOI stay was 0·8 days for the LiMAx^®^ group and 3·0 days for the control group (P < 0·001), representing a 73·3 per cent reduction in total IMCU/ICU days.

Patients in the LiMAx^®^ group had a shorter postoperative hospital stay than those in the control group (mean LOS 10·6 *versus* 13·3 days respectively; *P* = 0·012).

### Overall complication rate and mortality

The rate of severe complications was significantly lower in the LiMAx^®^ group than in the control group (14 (95 per cent c.i. 6 to 25) *versus* 28 (17 to 41) per cent respectively; *P* = 0·022) (*Table* 3). No statistically significant differences were observed for grade I or II complications.

**Table 3:** Complications after liver surgery according to the Clavien–Dindo classification^29^, in patients with evaluable data

	LiMAx^®^ group (*n* = 58)	Control group (*n* = 57)	*P* *
No complications	22 (38)	17 (30)	0·428
Grade I	22 (38)	22 (39)	0·532
Grade II	6 (10)	2 (4)	0·163
Grade ≥ IIIa	8 (14)	16 (28)	0·022
95% c.i. (%)	6, 25	17, 41	

Values in parentheses are percentages.

* Cochran–Mantel–Haenszel test stratified for centre.

One patient in each study group died. The patient who died in the control group developed postoperative pulmonary embolism leading to terminal right heart failure on postoperative day 18. The patient in the LiMAx^®^ group developed myocardial infarction during surgery leading to intraoperative resuscitation and interruption of liver resection; the patient subsequently developed irreversible right heart failure. One patient in each group developed posthepatectomy liver failure.

## Discussion

The use of liver function tests before and immediately after surgery resulted in significantly more patients being sent to the surgical ward after liver resection. Subsequently, the severe complication rate was lower, and LOI stay and LOS were significantly shorter in these patients.

The decision also to use the LiMAx^®^ test after surgery in the intervention arm was made for several reasons, including the frequency of both change in operation plans and additional intraoperative hepatic injury owing to intraoperative bleeding, inflow occlusion or less perfused resection margins. The primary endpoint of primary transfer to a general ward was chosen as this is a simple and comprehensive marker involving all preoperative, intraoperative and postoperative parameters of patient management, including residual liver function. The control arm revealed the current, rather conservative, standard of care in Germany, with postoperative telemetry in almost all patients having liver surgery. In contrast, most patients in the LiMAx^®^ arm had non‐critical residual liver function (LiMAx^®^ value above 150 μg per kg per h), and the majority was managed successfully by direct transfer to a general ward. No patient in the LiMAx^®^ group needed to be readmitted to the IMCU/ICU after the primary general ward transfer, and no readmissions were reported within 30 days after surgery.

The lower rate of severe complications in the LiMAx^®^ group was interesting. This may be explained by the individual preoperative volume–function analysis performed according to the LiMAx^®^ algorithm to preserve sufficient residual liver function^3^. The LiMAx^®^ protocol may result in surgeons being more aware of potentially impaired liver function. Subsequently, they may have adjusted the intraoperative resection strategy, surgical techniques or procedures. Identifying high‐risk patients for transfer to the IMCU/ICU according to the postoperative LiMAx^®^ value might have also allowed for prevention of some later severe complications by closer patient monitoring and optimal management. Various enhanced recovery after liver surgery (ERAS) protocols have been reported as safe and effective in optimizing treatment outcomes without compromising morbidity or mortality rates^30^, ^31^, ^32^, ^33^, ^34^. These protocols focus mainly on patient education, and early oral intake and mobilization. The present study protocol did not directly change perioperative or postoperative care elements, but simply stratified patients according to their residual liver function. A prompt postoperative referral to a general ward, however, indirectly triggered typical ERAS elements. In the ICU setting, nasogastric tubes, urinary catheters, arterial and central venous lines are usually kept inserted, mobilization of patients is restricted, and oral feeding is delayed^35^. In contrast, patients on a general ward routinely receive oral feeding and early mobilization as soon as possible, which is known to be a crucial factor in preventing postoperative complications^36^. Although such data were not collected explicitly, the present results suggest that patients did receive earlier oral feeding and/or mobilization after being sent directly to a general ward, according to common clinical practice in most liver centres.

The study has several limitations. Patients with complex liver resections including biliary or vascular reconstructions, and patients with previous resections or pre‐existing fibrosis or cirrhosis, were excluded from participation, even though such patients might derive even greater benefit from perioperative liver function assessment by the LiMAx^®^ test. The risk of severe postoperative complications, particularly PHLF, is expected to be much higher for patients with complex liver resections than in the investigated population^2^ ^6^, ^37^. The number of IMCU/ICU admissions in the control group was very high. In view of the present results, the conservative transfer policy might be changed in future.

The LiMAx^®^ test helps to transfer the patient to the right setting after liver surgery. Other factors, such as perioperative bleeding, spontaneous breathing, haemodynamic stability and adequate analgesia, are important, however, to guide postoperative transfer decision.

## Collaborators

The following are members of the Collaborative Fast‐track Liver Study Group: J. Pratschke, S. Chopra and G. Hunold (Charité – Universitätsmedizin Berlin, Berlin, Germany); J. Klempnauer, K. Timrott, S. Cammann, M. D. Jäger, B. Kettler, M. Kleine, W. Knitsch, U. Kulik, F. Oldhafer, W. Ramackers, C. Schumacher and L. Kousoulas (Hannover Medical School, Hannover, Germany); U. Settmacher, A. Nikolic, A. Bauschke, M. Gampe, H. Scheuerlein, A. Koch, V. Mategakis, L. Mutwol, U. Schöne, F. Uteß and F. Rauchfuß (University of Jena, Jena, Germany); M. Bartels, J. Broschewitz, J. Bucher, K. Mankel, G. Wiltberger, C. Benzing, L. Feldbrügge, M. Schmelzle, M. Schönberg and K. Semmling (University Hospital Leipzig, Leipzig, Germany), H. Lang, S. Heinrich, T. Huber, K. Alghdban and M. Kochergin (Johannes Gutenberg University, Mainz, Germany); C. T. Germer, F. Anger and U. Steger (University Hospital of Würzburg, Würzburg, Germany).
